# Volatile Employment and Health Insurance Gaps Among Low- and Middle-Income Workers

**DOI:** 10.1001/jamanetworkopen.2026.33883

**Published:** 2026-09-15

**Authors:** Vineeth Amba, Renuka Tipirneni, John Z. Ayanian, Sumit Agarwal

**Affiliations:** 1 Department of Medicine, Mount Sinai Hospital, New York, New York; 2 Department of Internal Medicine, University of Michigan Medical School, Ann Arbor; 3 Department of Health Management & Policy, University of Michigan School of Public Health, Ann Arbor; 4 Institute for Healthcare Policy and Innovation, University of Michigan, Ann Arbor

## Abstract

This cross-sectional study uses nationally representative data to assess employer-sponsored insurance offers and coverage among low- and middle-income workers with volatile employment in the US.

## Introduction

Many low-income individuals work in unstable or volatile jobs; in 2023, half of low-income households reported unpredictably fluctuating earnings.^1,2^ Research has shown certain workers (eg, part-time, blue-collar, temporary) have lower employer-sponsored insurance (ESI) offer and coverage rates.^3,4,5,6^ These studies extend only to 2017, and less evidence exists on the association between volatile employment, based on longitudinal work hours or earnings, and health coverage. New evidence could help inform expectations of the 2025 budget reconciliation bill (House Resolution 1) that will condition Medicaid eligibility on documented work hours and the December 2025 expiration of enhanced Advanced Premium Tax Credits (APTCs) which reduced Patient Protection and Affordable Care Act (ACA) marketplace affordability.^7^ Using nationally representative data, we assessed ESI offers and insurance coverage among low- and middle-income workers with volatile employment.

## Methods

For this cross-sectional study, we analyzed the 2022 to 2023 Medical Expenditure Panel Survey (MEPS) household component.^8^ Our sample consisted of adults aged 18 to 64 years who had a household income less than or equal to 400% of the federal poverty level and were employed in at least 1 round (eMethods in Supplement 1). Outcomes were a binary ESI offer measure and a categorical insurance coverage (ESI, Medicaid, marketplaces, other, and none) measure, assessed during the last round of 2023.

Leveraging the longitudinal MEPS design, we constructed 3 volatile employment measures: income volatility, work-hour volatility, and employment disruption. Income volatility was defined as a greater than 25% change in annual wage income from 2022 to 2023, relative to the average across both years.^9^ Work-hour volatility was defined as the within-person coefficient of variation of weekly hours worked across rounds, dichotomized into high and low volatility.^2^ Employment disruption was defined as any transition from employed to unemployed between consecutive rounds.^10^

We examined cross-sectional associations between volatile employment and ESI offer using binary logistic regression and between volatile employment and insurance coverage using multinomial logistic regression. Estimates reflect average marginal effects in percentage-point (pp) differences. Adjusted models accounted for age, sex, self-reported race or ethnicity, marital status, and presence of children. Statistical tests were 2-tailed (*P* < .05). We incorporated MEPS survey weights to account for the complex survey design and produce nationally-representative estimates. The study used public, deidentified data and was not considered human subjects research in accordance with 45 CFR §46. This study adhered to Strengthening the Reporting of Observational Studies in Epidemiology (STROBE) reporting guidelines. Analyses were conducted in Stata version 19.5 (StataCorp) from April 2025 to July 2026.

## Results

The study included 943 workers (representing 14.6 million individuals) with income volatility, 739 workers (11.4 million) with work-hour volatility, and 186 workers (2.9 million) who experienced employment disruption. Compared with those in stable work arrangements, workers with income volatility, work-hour volatility, and employment disruption were younger, likelier to be female, and had lower income (Table 1). Workers across all 3 volatility measures worked a mean of at least 30 hours per week when employed. Work hours fluctuated above and below 20 hours per week for 6.6 million (45.3%), 8.0 million (70.4%), and 2.4 million (82.7%) of income-volatile, hours-volatile, and employment-disrupted workers, respectively, compared with 1.4 million (13.3%), 0, and 5.6 million (25.1%) among their stable counterparts (Table 1).

**Table 1.** Demographic Characteristics of Low- and Middle-Income Workers in Volatile Employment Arrangements, Medical Expenditure Panel Survey, 2023^a^

Characteristic	Income volatility, % (95% CI)		Work hour volatility, % (95% CI)		Employment disruption, % (95% CI)	
	Volatile	Stable	Volatile	Stable	Volatile	Stable
Weighted population, (millions), No. (95% CI)^b^	14.6 (13.2-16.0)	10.7 (9.5-12.0)	11.4 (10.2-12.6)	13.5 (12.1-15.0)	2.9 (2.3-3.6)	22.4 (20.4-24.4)
Sample population, No.	943	678	739	857	186	1435
Age category						
18-29 y	35.2 (31.1-39.4)	21.8 (17.7-26.5)	43.4 (38.8-48.2)	17.8 (14.3-21.8)	49.2 (39.1-59.3)	26.9 (23.9-30.1)
30-44 y	33.9 (30.2-37.7)	45.4 (40.6-50.3)	31.7 (28.0-35.6)	44.2 (39.2-49.3)	23.0 (15.9-32.2)	40.8 (37.5-44.2)
45-54 y	17.2 (14.3-20.5)	18.6 (15.4-22.3)	13.4 (10.6-16.8)	21.8 (18.8-25.0)	12.6 (7.6-20.2)	18.5 (16.1-21.0)
55-64 y	13.8 (11.3-16.9)	14.2 (11.4-17.7)	11.6 (9.1-14.5)	16.3 (13.3-19.7)	15.2 (10.2-22.1)	13.8 (11.7-16.2)
Sex						
Female	50.3 (46.7-53.9)	46.5 (42.1-51.0)	54.0 (49.3-58.7)	44.1 (40.3-48.0)	52.7 (43.1-62.2)	48.1 (45.3-51.0)
Male	49.7 (46.1-53.3)	53.5 (49.0-57.9)	46.0 (41.4-50.8)	55.8 (51.9-59.7)	45.7 (36.5-55.2)	52.1 (49.2-54.9)
Race or ethnicity^c^						
Hispanic	30.8 (25.5-36.7)	28.6 (22.9-35.1)	29.0 (24.0-34.6)	30.8 (25.2-37.0)	22.7 (14.5-33.8)	30.8 (25.8-36.2)
Non-Hispanic Black	18.4 (14.4-23.2)	16.8 (13.2-21.2)	19.6 (15.4-24.7)	16.1 (12.8-20.0)	20.5 (13.9-29.1)	17.4 (14.1-21.2)
Non-Hispanic White	42.4 (37.8-47.2)	48.2 (42.0-54.5)	41.9 (37.1-46.8)	47.6 (42.1-53.1)	48.2 (38.5-57.9)	44.5 (40.0-49.0)
Non-Hispanic other race	8.4 (6.2-11.4)	6.4 (4.0-9.8)	9.5 (7.0-12.8)	5.6 (3.8-8.2)	8.6 (4.5-16.1)	7.4 (5.5-10.0)
Annual 2023 income (% FPL)						
0% to 138% of the FPL	32.6 (28.8-36.7)	14.6 (11.7-18.1)	35.2 (31.1-39.6)	16.0 (13.1-19.6)	52.8 (43.5-62.0)	21.3 (18.8-24.0)
250% to 400% of the FPL	31.6 (26.9-36.6)	48.7 (43.5-54.0)	29.2 (24.5-34.5)	47.2 (42.2-52.2)	16.5 (9.9-26.2)	41.8 (37.6-46.0)
Insurance category, %						
Employer-sponsored insurance	36.7 (32.3-41.3)	58.1 (53.0-63.0)	35.4 (30.7-40.5)	55.5 (50.7-60.3)	24.5 (16.2-35.1)	48.5 (44.6-52.5)
Medicaid	29.2 (25.2-33.6)	15.9 (12.5-20.0)	31.2 (26.8-35.9)	16.4 (13.2-20.2)	42.8 (33.2-53.0)	21.1 (18.1-24.4)
Marketplaces	9.7 (7.0-13.2)	5.2 (3.5-7.8)	9.0 (6.1-13.0)	6.6 (4.7-9.1)	9.6 (4.7-19.0)	7.5 (5.5-10.2)
Other	6.0 (4.1-8.9)	4.3 (2.4-7.6)	6.3 (4.3-9.0)	4.6 (2.7-7.5)	4.8 (2.3-9.9)	5.4 (3.8-7.5)
Uninsured	18.4 (15.1-22.3)	16.5 (13.1-20.4)	18.2 (14.6-22.4)	17.0 (13.9-20.7)	18.3 (11.7-27.3)	17.5 (14.7-20.7)
Married	34.9 (30.4-39.7)	38.0 (32.6-43.8)	27.1 (22.4-32.3)	44.2 (38.9-49.5)	25.6 (17.0-36.5)	37.6 (33.6-41.9)
Have children	40.3 (35.6-45.2)	47.2 (42.1-52.3)	32.7 (28.2-37.6)	52.8 (47.8-57.7)	30.5 (21.8-40.8)	44.9 (40.9-48.9)
Hours worked per week during employment, mean (95% CI)	34.4 (33.4-35.3)	38.1 (37.0-39.1)	33.4 (32.4-34.5)	38.0 (37.1-38.9)	32.8 (30.3-35.3)	36.3 (35.6-37.1)
Hours fluctuated above and below 20 h/wk across rounds	45.3 (41.2-49.5)	13.3 (10.4-17.0)	70.4 (66.0-74.6)	0	82.7 (72.5-89.6)	25.1 (22.3-28.1)

Abbreviation: FPL, federal poverty level.

^a^ All characteristics reflect values at round 5 (approximately July to December 2023) aligning with the timing of the primary study outcomes, with the exception of the hours worked per week variable, which reflects a mean across all rounds in which the participant was actively employed. Volatility measures are not mutually exclusive. Thus, a participant could be classified as volatile in more than measure.

^b^ Weighted estimates are representative of the civilian, noninstitutionalized US population.

^c^ Race and ethnicity variables were self-reported by Medical Expenditure Panel Survey participants. Non-Hispanic other race includes non-Hispanic Alaskan Native or American Indian, non-Hispanic Asian or Pacific Islander, and non-Hispanic Multiracial.

In adjusted analyses, workers with income volatility and work-hour volatility were significantly less likely to be offered ESI compared with their stable counterparts by 14.9 pp (95% CI, −21.1 to −8.7 pp) and 14.4 pp (95% CI, −21.1 to −7.7 pp), respectively (Table 2). Workers with employment disruption were also less likely to be offered ESI by 18.9 pp (95% CI, −42.4 to 4.7 pp), but this was statistically insignificant. All volatile work groups were significantly less likely to have ESI by 19.6 to 24.1 pp (all *P* < .001) and more likely to have Medicaid coverage by 12.2 to 18.9 pp (all *P* < .001). Workers with income and work-hour volatility were 5.0 pp (95% CI, 1.7 to 8.3 pp) and 4.3 pp (95% CI, 0.4 to 8.2 pp) more likely to have ACA marketplace coverage than their stable counterparts.

**Table 2.** Associations Between Employment Volatility Type in Low- and Middle-Income Workers and Being Offered ESI, Insurance Type^a^

Insurance coverage category	Income		Hours		Employment	
	Volatile	Stable	Volatile	Stable	Disrupted	Stable
Offer of ESI at current main job (vs no offer)^b^						
Estimated probability, %	53.9	68.9	52.3	66.6	42.8	61.7
Difference, pp (95% CI)	−14.9 (−21.1 to −8.7)	[Reference]	−14.4 (−21.1 to −7.7)	[Reference]	−18.9 (−42.4 to 4.7)	[Reference]
*P* value	<.001	NA	<.001	NA	0.12	NA
ESI						
Estimated probability, %	37.0	57.6	35.6	55.2	24.4	48.6
Difference, pp (95% CI)	−20.5 (−26.3 to −14.8)	[Reference]	−19.6 (−26.4 to −12.8)	[Reference]	−24.1 (−33.4 to −14.9)	[Reference]
*P* value	<.001	NA	<.001	NA	<.001	NA
Medicaid						
Estimated probability, %	28.6	16.5	29.4	17.2	40.1	21.2
Difference, pp (95% CI)	12.2 (7.1 to 17.2)	[Reference]	12.2 (6.8 to 17.6)	[Reference]	18.9 (9.0 to 28.7)	[Reference]
*P* value	<.001	NA	<.001	NA	<.001	NA
Marketplace						
Estimated probability, %	10	5.0	10.3	5.9	10.5	7.4
Difference, pp (95% CI)	5.0 (1.7 to 8.3)	[Reference]	4.3 (0.4 to 8.2)	[Reference]	3.1 (−4.5 to 10.6)	[Reference]
*P* value	0.003	NA	0.03	NA	0.42	NA
Other						
Estimated probability, %	5.6	4.8	5.2	5.5	3.6	5.6
Difference, pp (95% CI)	0.8 (−2.8 to 4.3)	[Reference]	−0.4 (−3.8 to 3.0)	[Reference]	−2.0 (−5.1 to 1.2)	[Reference]
*P* value	0.52	NA	0.83	NA	0.22	NA
Uninsured						
Estimated probability, %	18.8	16.1	19.5	16.1	21.3	17.2
Difference, pp (95% CI)	2.6 (−1.6 to 6.9)	[Reference]	3.4 (−1.7 to 8.6)	[Reference]	4.1 (−4.0 to 12.3)	[Reference]
*P* value	0.22	NA	0.19	NA	0.32	NA

Abbreviations: ESI, employer-sponsored insurance; NA, not applicable; pp, percentage point.

^a^ Survey weights were applied to all regression models to ensure estimates were representative of the civilian, noninstitutionalized US population. All results are from a multivariable model that controlled for age, sex, self-reported race or ethnicity (using mutually exclusive categories), marital status, and presence of children in household. Estimates are reported as average marginal effects to facilitate interpretation of coefficients as absolute percentage-point differences.

^b^ Among self-employed respondents, the offer of ESI was assessed in those who were self-employed with at least 1 other employee.

## Discussion

Low- and middle-income workers with volatile employment were substantially less likely to be offered or be covered by ESI, and more likely to have Medicaid or ACA marketplace coverage, underscoring the importance of nonemployer-based coverage for this population. Yet under House Resolution 1, Medicaid eligibility will be conditioned on an 80-hour monthly work requirement and more frequent eligibility redeterminations. Therefore, workers with volatile employment will be particularly susceptible to Medicaid disenrollment by nature of their fluctuating hours and employment—supported by our finding that these workers were more likely to have hours crossing above and below 20 hours per week.^2,7^ Notably, workers across all 3 volatility measures worked a weekly average of at least 30 hours when employed, suggesting Medicaid disenrollment may reflect challenges meeting documentation requirements designed around stable employment rather than insufficient work hours. Moreover, the enhanced APTC expiration may reduce ACA marketplace coverage affordability, further jeopardizing coverage options.^7^

Limitations include self-reported data and the contemporaneous unwinding of continuous Medicaid coverage, which may influence generalizability. These findings highlight the importance of protecting coverage pathways for workers in volatile work arrangements with limited ESI access.

## Notes

Supplement 1. eMethods.

Supplement 2. Data Sharing Statement
