# Supplementary material for: Volatile Employment and Health Insurance Gaps Among Low- and Middle-Income Workers
Source: JAMA Netw Open. 2026 Sep 15;9(9):e2633883. doi: 10.1001/jamanetworkopen.2026.33883 (PMC13579437; doi:10.1001/jamanetworkopen.2026.33883)
Supplement: Supplementary file 1 [file jamanetwopen-e2633883-s001.pdf]

Supplemental Online Content

Amba V, Tipirneni R, Ayanian JZ, Agarwal S. Volatile employment and health insurance gaps among low- and middle-income workers. *JAMA Netw Open.* 2026;9(9):e2633883. doi:10.1001/jamanetworkopen.2026.33883

eMethods

This supplemental material has been provided by the authors to give readers additional information about their work.

© 2026 Amba V et al. *JAMA Network Open*.

eMethods

Data Source

We used data from Panel 27 of the Medical Expenditure Panel Survey (MEPS) Household Component, a nationally representative longitudinal survey of the non-institutionalized, civilian United States (US) population by the Agency for Healthcare Research and Quality (AHRQ). Panel 27 follows a cohort of individuals across five rounds of interviews ranging from Quarter 1 of 2022 to Quarter 4 of 2023. Data were accessed through IPUMS Health Surveys.[1] Panel 27 was chosen as it was the most recent complete panel of data available.

Analytical Sample

We applied the following inclusion and exclusion criteria to Panel 27 of the MEPS. We included adults aged 18-64 years who had a household income ≤ 400% of the Federal Poverty Level and were employed in at least one round. We excluded individuals with Medicare, CHAMPUS, TRICARE, or CHAMP-VA coverage. Our final sample included 1,621 individuals represented in all five rounds of the panel.

Outcome Variables

Offer of employer-sponsored insurance (ESI) was defined as a binary indicator of whether the respondent's current main job offered ESI. This outcome was assessed in Round 5, representing approximately Quarter 3 and Quarter 4 of 2023.

Insurance coverage category was created from MEPS monthly insurance variables between July to December 2023, also representing Quarter 3 and Quarter 4 of 2023. This timeframe was chosen to avoid overlap with the Medicaid continuous enrollment period (which ended on March 31, 2023). For each individual, health insurance type was assigned based on whether they held that coverage type in any month in the six-month window spanning Round 5. Coverage was assigned hierarchically: 1) ESI, 2) Medicaid, 3) Marketplace, 4) Uninsured, 5) Other. The "Other" category included uncategorized insurance and other private insurance plans (eg direct-purchase plans).

Volatility Measures

Three volatile employment measures were constructed. Of note, in these measures of employment volatility, individuals may meet criteria for more than one measure.

Income volatility was defined as a greater than 25% change in annual wage income between 2022 and 2023, relative to the average of the two years of the panel. We derived our definition from the work of Dynan et al. which observes volatility bi-directionally (both increases and decreases in income when comparing years).[2] We applied the 25% threshold in either direction based on the magnitude used in work by Hacker et al.[3] Annual wage income was derived from a MEPS person- level income variable that represented income for the calendar year.

Work-hour volatility was defined as the within-person coefficient of variation of weekly hours worked across all panel rounds in study participants, following Bauer et al.[4] The coefficient of variation was calculated as the ratio of within-person standard deviation to the mean of weekly hours multiplied by 100, which was then dichotomized for comparisons of high and low volatility. Weekly hours were obtained from a round-specific hours worked variable. Those "not in universe" for this variable (reflect no current main job) were coded as having worked 0 hours.

Employment disruption was defined as any transition from employed to unemployed between consecutive panel rounds, following Agarwal et al.[5]

© 2026 Amba V et al. *JAMA Network Open*.

Covariates
Covariates were assessed in the same year and/or round as the outcomes. These included age (categorical), sex (male/female) as reported by participants, income as a percentage of the federal poverty level, and race and ethnicity. In regard to race and ethnicity, these variables were self-reported by MEPS participants. We used the race and ethnicity variables to create the mutually exclusive categories of: Hispanic, Non-Hispanic Black, Non-Hispanic White, and Non-Hispanic Other Race. As also noted in our Table 1 footnote, "Non-Hispanic Other Race" includes "Non-Hispanic Asian or Pacific Islander," "Non-Hispanic Alaskan Native or American Indian," and "Non-Hispanic Multiple Race." Round-specific variables included binary measures for being married and presence of children.

Statistical Analysis
We examined cross-sectional associations between volatile employment and ESI offer using binary logistic regression and between volatile employment and insurance coverage using multinomial logistic regression. Estimates are reported as average marginal effects to facilitate interpretation of coefficients as absolute percentage-point (pp) differences. Adjusted models accounted for age, sex, self-reported race/ethnicity, marital status, and presence of children. Statistical tests were 2-tailed with a significance level of $P < 0.05$. We incorporated MEPS survey weights (PERWEIGHT) to account for complex sample design, nonresponse, and pooling to produce national estimates.[1]

Literature Cited

1. Lynn A. Blewett, Julia A. Rivera Drew, Andrew Fenelon, Daniel Backman, Etienne Breton, Grace Cooper, Stephanie Richards, and Renae Rogers. IPUMS Health Surveys: Medical Expenditure Panel Survey, Version 3.0 [dataset]. Minneapolis, MN: IPUMS, 2025. https://doi.org/10.18128/D071.V3.0.
2. Dynan K, Elmendorf D, Sichel D. The Evolution of Household Income Volatility. *B.E. Journal of Economic Analysis & Policy*. 2012;12(2)
3. Hacker, J.S., Huber, G.A., Nichols, A., Rehm, P., Schlesinger, M., Valletta, R. and Craig, S. (2014), The Economic Security Index: A New Measure for Research and Policy Analysis. Review of Income and Wealth, 60: S5-S32. https://doi.org/10.1111/roiw.12053
4. Bauer L, East CN, Howard O. Low-income workers experience—by far—the most earnings and work hours instability. The Hamilton Project, Brookings Institution. January 9, 2025. Available at: https://www.hamiltonproject.org/publication/post/low-income-workers-experience-by-far-the-most-earnings-and-work-hours-instability/. Accessed July 9, 2026.
5. Agarwal SD, Sommers BD. Insurance Coverage after Job Loss - The Importance of the ACA during the Covid-Associated Recession. N Engl J Med. 2020;383(17):1603-1606. doi:10.1056/NEJMp2023312

© 2026 Amba V et al. *JAMA Network Open*.
