# Supplementary material for: Volatile Employment and Health Insurance Gaps Among Low- and Middle-Income Workers
Source: JAMA Netw Open. 2026 Sep 15;9(9):e2633883. doi: 10.1001/jamanetworkopen.2026.33883 (PMC13579437; doi:10.1001/jamanetworkopen.2026.33883)
Supplement: Supplementary file 2 [file jamanetwopen-e2633883-s002.pdf]

Data Sharing Statement

Amba. Volatile Employment and Health Insurance Gaps Among Low- and Middle-Income Workers. *JAMA Netw Open*. Published September 15, 2026.
doi:10.1001/jamanetworkopen.2026.33883

Data
Data available: Yes
Data types: Data (not involving human participants)
How to access data: https://meps.ipums.org/meps/
When available: With publication

Supporting Documents
Document types: None

Additional Information
Who can access the data: Anyone requesting data.
Types of analyses: For any purpose.
Mechanisms of data availability: With or without investigator support.
